# Hydraulic and photosynthetic responses of big sagebrush to the 2017 total solar eclipse

**DOI:** 10.1038/s41598-019-45400-y

**Published:** 2019-06-20

**Authors:** Daniel P. Beverly, Carmela R. Guadagno, Mario Bretfeld, Heather N. Speckman, Shannon E. Albeke, Brent E. Ewers

**Affiliations:** 1 Department of Botany, University of Wyoming, Laramie, WY 82071 USA; 2 Water Resources, Environmental Science and Engineering, University of Wyoming, Laramie, WY 82071 USA; 3 Department of Geography and Wyoming Geographic Information Service Center, University of Wyoming, Laramie, WY 82071 USA; 4 Program in Ecology, University of Wyoming, Laramie, WY 82071 USA

**Keywords:** Light responses, Ecophysiology

## Abstract

The total solar eclipse of August 21, 2017 created a path of totality ~115 km in width across the United States. While eclipse observations have shown distinct responses in animal behavior often emulating nocturnal behavior, the influence of eclipses on plant physiology are less understood. We investigated physiological perturbations due to rapid changes of sunlight and air temperature in big sagebrush (*Artemisia tridentata* ssp. *vaseyana*), a desert shrub common within the path of eclipse totality. Leaf gas exchange, water potential, and chlorophyll *a* fluorescence were monitored during the eclipse and compared to responses obtained the day before in absence of the eclipse. On the day of the eclipse, air temperature decreased by 6.4 °C, coupled with a 1.0 kPa drop in vapor pressure deficit having a 9-minute lag following totality. Using chlorophyll *a* fluorescence measurements, we found photosynthetic efficiency of photosystem II (*Fv’/Fm’*) recovered to near dark acclimated state (i.e., 87%), but the short duration of darkness did not allow for complete recovery. Gas exchange data and a simple light response model were used to estimate a 14% reduction in carbon assimilation for one day over sagebrush dominated areas within the path of totality for the Western United States.

## Introduction

The total solar eclipse of August 21, 2017 was one of the most anticipated astrological phenomena to date, inspiring over 150 million of people from all over the world to observe the rare event^1^. The *umbra*−inner region of the shadow cast by the moon with total occlusion of sunlight−spanned a width up to 115 km, with approximately 200 seconds of totality observed along the line of maxima. The *penumbra*−marginal region of the shadow cast by the moon with partial occlusion of sunlight−entered the continental United States at 17:19 UTC in Oregon and it was visible throughout the continent exiting in South Carolina at approximately 18:48 UTC^2^. Nearly 46 million hectares of land were affected by totality, sparking the most disparate conversations of the eclipse impacts, from animal behavior to the power grid^3,4^.

Studies on animal behavior during solar eclipses have shown mixed responses to solar eclipses. Birds, bees, and spiders seem to behave just as in dusk conditions; returning to roosts, altering foraging behavior, or dismantling webs^5–7^. In contrast, little to no behavioral change was observed in dairy cattle during grazing and in captive chimpanzees^8,9^.

Very little is known about plant and ecosystem responses to eclipses and the limited number of previous studies lack mechanistic descriptions. Plants cope with decreasing temperatures, changes in vapor pressure deficit (VPD), and more or less sudden variations of incoming radiation on a daily basis. These factors are considered primary drivers of photosynthesis and transpiration^10^ and their fluctuations have been shown to largely influence photochemical activity and evaporative demand^11–15^. Besides this exogenous control, photosynthesis and transpiration are also synced to the time of the day by the presence of an endogenous clock that responds to environmental cues including fluctuations of light^16,17^. The eclipse serves as an natural experiment that we can use to elucidate possible photochemical and hydraulic consequences from light stress and more generally to hypothesize on the response of plants to brief stress events. Generally, plants respond to simultaneous changes in more than one single driver and distinct responses can only be obtained *via* experimental manipulations. Not surprisingly, the only previous observations of a total solar eclipse at the plant or stand level showed a significant reduction of transpiration rates and increasing CO_2_ concentrations throughout crop canopies reflecting a decrease in photosynthesis while respiration continues^18–21^.

The path of totality of the 2017 eclipse covered a large percentage of the semi-arid shrub lands throughout the western United States (Fig. S1). Many of these communities are considered to be at risk given climatic uncertainty and habitat fragmentation due to urbanization and energy extraction^22–24^. We chose big sagebrush (*Artemisia tridentata* Nutt.) as our species of interest since it dominates semi-arid ecosystems, covering more than 160,000 *km*^2^ from Oregon to New Mexico^25^. Big sagebrush ecosystems support a regionally high species diversity and they represent key contributions to local biogeochemical cycles, with the majority of stored carbon sequestered in above- and belowground woody biomass and soil organic matter^26,27^. These distinct communities are characterized by physiological adaptations to semi-arid environments, including hydraulic redistribution of deep ground water reserves and canopy architecture allowing for maximal water-use efficiency^13,28–30^. Additionally, transpiration controls are governed by endogenous clocks that are sensitive to environmental cues including fluctuations of light^16,17^. The short-term stress imposed by the eclipse serves as natural experiment to elucidate photochemical and hydraulic shock and recovery to light-stress events^16,31^. Further, solar eclipses represent an unexpected event for plants, potentially causing sudden changes in gene expression to cope with the new environment. The resonance between the external cues and internal circadian rhythms has a clear effect on plant performance^32–35^. Any transcriptomic change can result in a cascade of events leading to downstream physiological adjustments from the immediate tuning of stomatal aperture to more specific mechanisms of light capture and utilization, regulation of carboxylation rates, or regulating sugar metabolism^36–39^. Various stresses from drought to extreme temperature and salinity share pathways of response in plants due to similar osmotic adjustments and oxidative burst caused by these strains^38^. Being unexpected and short in duration, we can assume the rapid diminishing of incoming radiation during the solar eclipse is a stressful and shock event with a potential decrease in plant photosynthetic and gas exchange performance. Under natural conditions, hydraulic and photosynthetic mechanisms may require minutes to hours to recover to pre-stress levels with the limits of photoprotective mechanisms and significance of photoinhibition still under investigation^40–43^.

Here, we hypothesize that solar eclipses instigate a rapid but progressive light limitation, similar to the one experienced by leaves measured for light response curves during controlled chamber experiments^44^. We tracked the sudden change in radiation, temperature, and VPD and analyzed the consequences on leaf physiological traits. We focused our attention on the possible changes in the efficiency of photosystem II (PSII). In particular, we followed the dynamics of the maximum efficiency of PSII, in both dark (*F*_*v*_/*F*_*m*_) and light conditions (*F*_*v*_*’*/*F*_*m*_*’*), and the non-photochemical quenching (NPQ) of chlorophyll *a* fluorescence as proxies of acclimation to progressing light fluctuation and possible stress response^45–48^. We also analyzed leaf water potential (Ψ_*L*_) and leaf transpiration (*E*_*L*_) as mechanistic components of hydraulic status during the eclipse with particular attention to rates of recovery during the stress event. We compared the dynamics for each physiological trait during the eclipse to the dark-acclimated predawn state to capture significant perturbations and to elucidate possible underpinning mechanisms. Finally, we used a first principles approach to estimate how the eclipse impacted total carbon assimilation from big sagebrush-dominated ecosystems within the path of totality across the United States.

Despite its relatively short duration (~2 hrs.), the eclipse caused significant reduction in estimated daily carbon simulation rates for August 21, 2017 in big sagebrush ecosystems. Our results use chlorophyll *a* fluorescence and hydraulic traits for model implantation towards a more comprehensive understanding of plant physiological responses to sudden perturbations in light, temperature, and humidity that the internal clock fails to predict.

## Results

**Figure 1:**
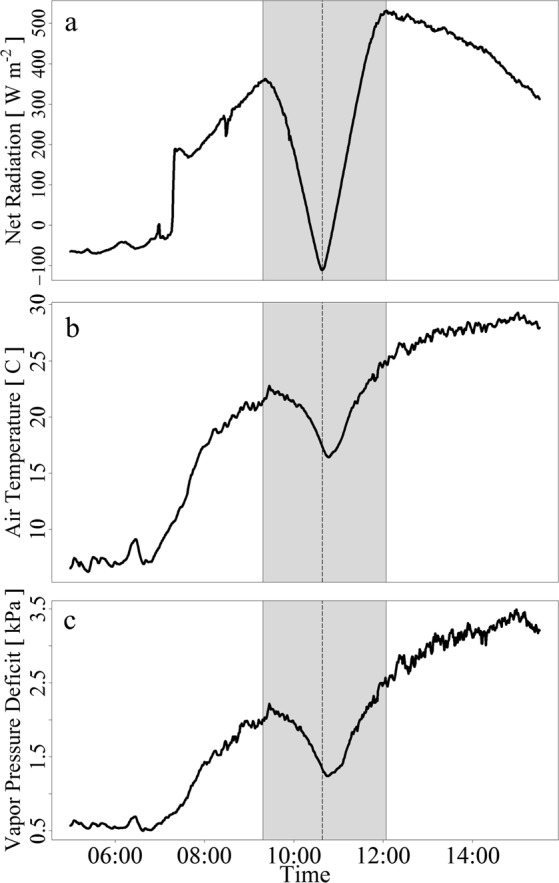
Time series of environmental factor responses from the total solar eclipse (August 21, 2017). Net radiation (**a**) reached −111*Wm*^−2^ minimum during totality. Temperature (**b**) and vapor pressure deficit (**c**) decrease to midday minimum 9 minutes post totality. Gray shaded areas represent partial soil eclipse. Dashed vertical line represents total solar eclipse.

The day of the eclipse had nearly zero cloud cover; minimal radiation inputs during the solar eclipse occurred during totality with net radiation of −111 *Wm*^−2^ and PPFD of 0.0 *μmolm*^−2^*s*^−1^ (Fig. 1a). With an air temperature decrease of 32.7% and a 41.0% increase in humidity at totality, the eclipse caused a 1.0 kPa drop in VPD from 2.2 to 1.2 kPa (Fig. 1b,c). As expected, the eclipse caused instantaneous changes in radiation while the disruptions in temperature and VPD lagged behind totality by 9-minutes (Fig. 1).

**Figure 2:**
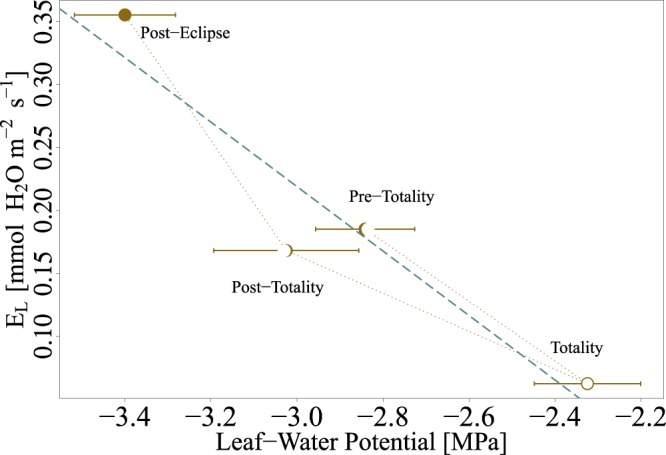
Hysteresis of transpiration (*EL*) with respect to leaf-water potential (Ψ *L*) pre-, during, and post-totality then post-partial eclipse. Error bars represent the standard error (*n* = 6). Whole-plant conductivity (*KL*) was estimated using linear regression (green).

Average predawn Ψ_*L*_ for the day of and day prior to the eclipse were not significantly different, −2.0 ± 0.2 and −1.9 ± 0.4 MPa, respectively, and suggests substantial soil-water limitation during measurements. The day before the eclipse average midday Ψ_*L*_ was −4.3 ± 0.4 MPa, whereas midday Ψ_*L *_on day of the eclipse, after totality, was −3.4 ± 0.1 MPa (Table 1). Thirty-minutes prior to totality, Ψ_*L*_ was 33.0% lower than predawn Ψ_*L*_ (i.e., −2.8 ± 0.1 MPa) (Fig. 2; Table 1). Seconds following totality, Ψ_*L*_ recovered to within 9.1% from predawn Ψ_*L*_ (−2.3 ± 0.1 MPa; Fig. 2; Table 1). Time series measurements of (*E*_*L*_), from the LiCor gas analyzer, declined 70% during the solar eclipse (Figure S2) and stomatal conductance calculated from *E*_*L*_ declined from 0.012 to 0.002 *mol* H_2_O *m*^−2^*s*^−1^. The change in *g*_*s*_ followed the 9-minute lag in VPD following totality. Whole-plant conductivity on the day of the eclipse was 0.256 *mmol* H_2_O *m*^−2^*s*^−1^*MPa*^−1^ (0.46 *10^−3^ *g* H_2_O *m*^−2^*s*^−1^*MPa*^−1^) (Fig. 2). Throughout the day of the eclipse, leaf-water potentials significantly correlated with leaf-level transpiration rates at the time of sampling (*R*^2^ = 0.84, *p-value* < 0.05), inferring no lag in gas exchange and hydraulic response, thus capacitance was negligible as expected for small desert shrubs. Albeit not explicitly tested, variability of canopy Ψ_*L*_ was assumed to be low with minimal variation observed within time point replication (Fig. 2).

**Table 1:** Leaf-water potential (Ψ_*L*_) for the day prior and day of the eclipse. Error represented by standard error of samples for each time point (*n* = 6).

Time MST	Leaf-water potential (Ψ_*L*_) MPa
8/20/2017 5:00	_−1.9 ± 0.4 *α*_
8/20/2017 10:00	_−3.5 ± 0.1 *cd*_
8/20/2017 15:15	_−4.3 ± 0.4 *d*_
8/20/2017 19:25	_−3.6 ± 0.2 *cd*_
8/21/2017 5:00	_−2.0 ± 0.2 *α*_
8/21/2017 10:00	_−2.8 ± 0.1 *abc*_
8/21/2017 10:40	_−2.3 ± 0.1 *ab*_
8/21/2017 11:25	_−3.0 ± 0.2 *abc*_
8/21/2017 12:30	_−3.4 ± 0.1 *bcd*_

Letters indicate significant differences between time point measurements.

**Figure 3:**
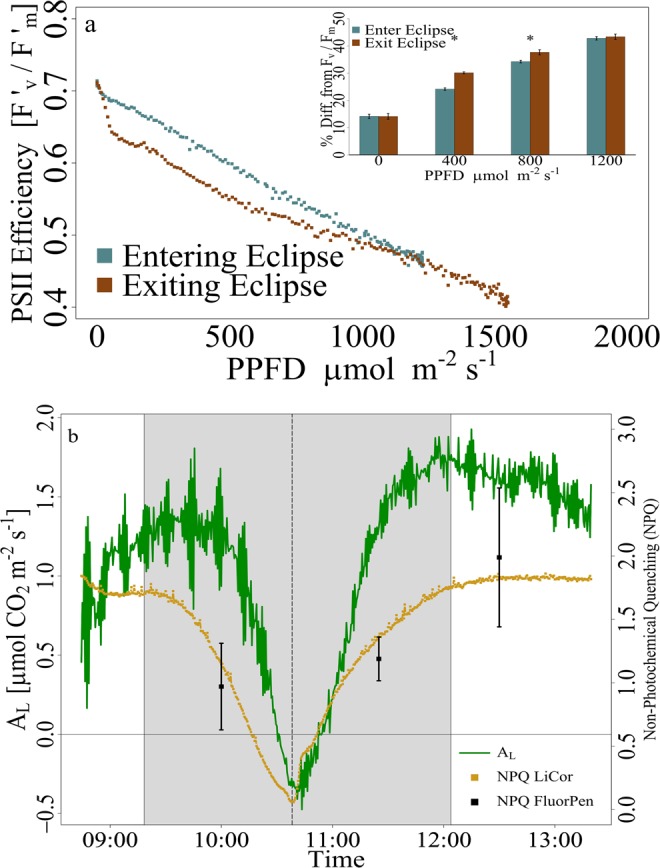
Hysteresis of PSII efficiency with respect to PPFD (**a**) entering (blue) and exiting (red) the solar eclipse with perturbations of PSII after totality. Inset of entering and exiting percent differences relative to dark-acclimated PSII efficiency (*Fv*/*Fm*) recovering under high light. Stars denote significant differences (p < 0.05) in light levels between entering and exiting the eclipse. Big sagebrush observed declines in CO_2_ assimilation rates (*A*_*L*_, green) with reduced radiation (**b**). Non-photochemical quenching (NPQ) of Fluorpen (black squares) and LiCor fluorometer (yellow) measurements recorded every 30 seconds throughout the eclipse decline until totality (dotted) and recovery to pre-eclipse conditions. Error bars represent standard error (*n* = 6). Gray shaded areas represent partial soil eclipse. Dashed vertical line represents total solar eclipse.

Prior to the eclipse, using pulses from the LiCor fluorometer, PSII efficiency in light conditions (*F*_*v*_*’*/*F*_*m*_*’*) was 0.46 and it followed a linear recovery towards a dark-acclimated PSII efficiency (*F*_*v*_/*F*_*m*_; 0.81) during the solar eclipse (Fig. 3a). As sunlight decreased (i.e., pre-totality), PSII efficiency from Licor fluorometer (i.e., *F*_*v*_*’*/*F*_*m*_*’*) recovered to a maximum of 0.71, a 13.2% difference from the dark-acclimated state (*F*_*v*_/*F*_*m*_) prior to sunrise (Fig. 3a). During increasing light incidence (i.e., post-totality), PSII efficiency exhibited a hysteresis pattern with respect to PPFD until new high light (i.e., 1200 *μmolm*^−2^*s*^−1^) conditions. Time series measurements of NPQ, calculated from LiCor fluorometer, progressively decreased from 1.83 to 0.06 during totality finally recovering to 1.83 after partial eclipse. Data from the replicated hand-held fluorometer illustrated similar photochemical responses with NPQ increasing 24% from an average of 0.76 ± 0.34 to 1.08 ± 0.55 following totality (Fig. 3b).

The empirically modeled light-response of carbon assimilation was strongly driven (*R*^2^ = 0.92) by PPFD (Fig. S2). Modeled carbon assimilation assuming no eclipse and the observed carbon assimilation during the eclipse resulted in a difference of 266.7 *μmol* CO_2_ *m*^−2^*s*^−1^ over 2 hours 45 minutes 28 seconds, over duration of the eclipse, from partial to total (Fig. 3b).

**Figure 4:**
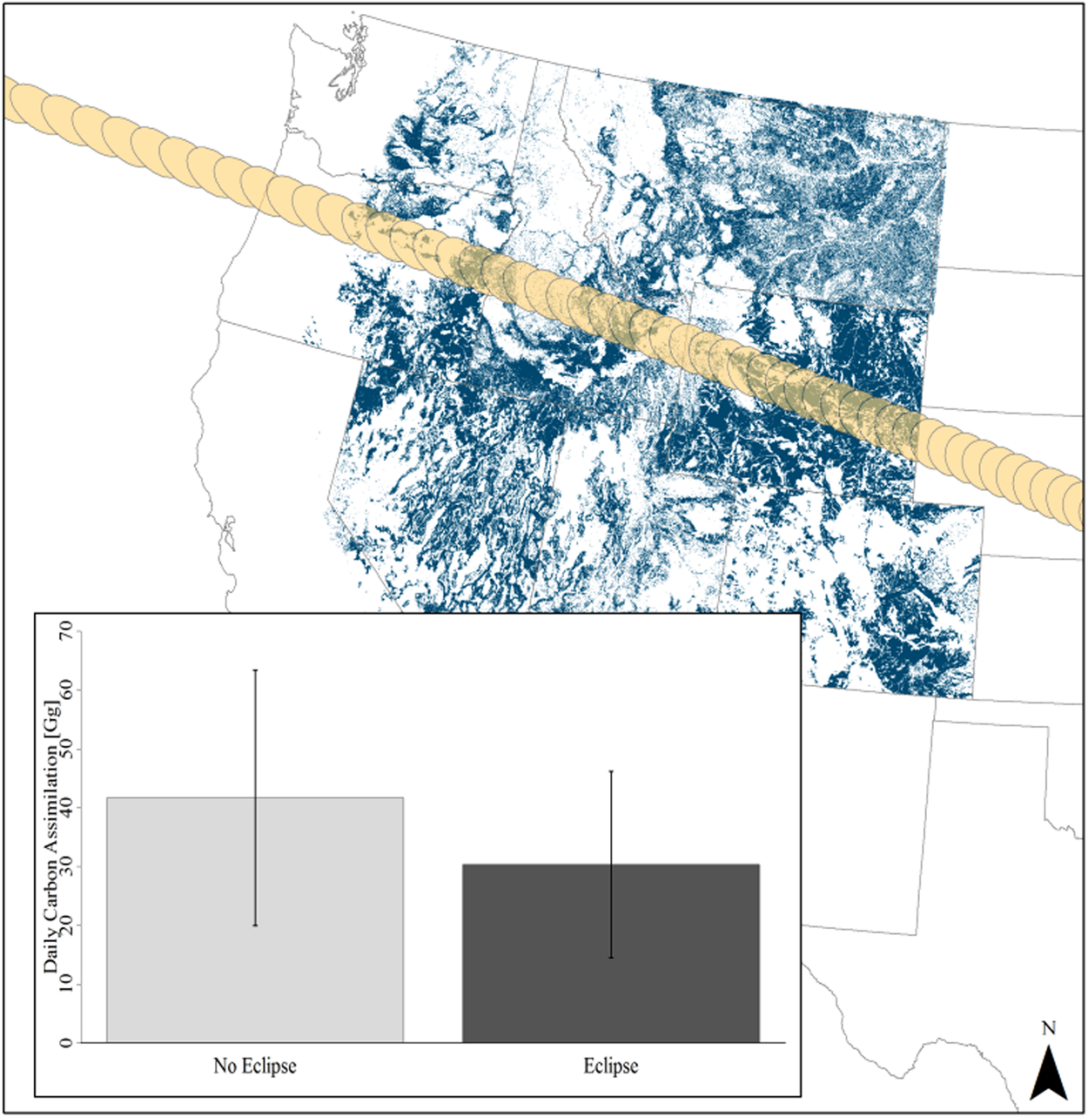
Map of big sagebrush distributions (blue) across the western United States. Big sagebrush distributions within the eclipse *umbra* (gold) were scaled and modeled given no eclipse and under observed eclipse assimilation rates. Inset shows model results throughout the range, with the eclipse resulting in a 14% reduction in daily CO_2_ assimilation. Error bars are representative of upper and lower bounds of carbon assimilation for leaf area and stand density for stands of 25–40 years of age.

There was a significant relationship (*R*^2^ = 0.67, F = 74.8, *p-value* < 0.001) between fine resolution shrub and coarse resolution sagebrush data, allowing for extrapolation of sagebrush distributions across the species native range. Modeled assimilation rates from light response and observed assimilation rates were used to estimate reductions of carbon assimilation for the total big sagebrush leaf area of 14710.9 ± 5658.0 *km*^2^ *km*^−2^ throughout distributions within the path of totality. Uncertainty within leaf area measurements were scaled from the assumptions of pixel densities, age, and stand density allometrics. The difference suggests a 14% reduction in assimilation due to the solar eclipse, from 41.3 ± 21.6 Gg assimilated carbon assuming no eclipse to 35.9 ± 18.6 Gg assimilated for the day of the total solar eclipse (Fig. 4).

## Discussion

Reduced temperature, VPD, and especially incoming radiation, shocked sagebrush circadian clock triggering a response far beyond typical cloud effects, with a consequent decline in photosynthesis and transpiration (Figs 1–3)^49^. However, the duration of totality was not sufficient to restore a physiological dark-acclimated state of PSII in comparison to predawn/pre-stress conditions.

**Leaf-Level response** from measurements of PSII efficiency (LiCor) at totality reached values close (i.e., within 87%) to dark-acclimated conditions (Fig. 3a). The sudden decrease of PSII efficiency and NPQ upon limiting light supports that light harvesting complexes are capable of adjusting in very short time, but the responses were not quick enough under the dynamic light conditions of the solar eclipse^17,50^. Similar to shade-adapted leaves and sunflecks, the canopy acclimates quickly to high energy with changes of photochemical regulation towards energy dissipation mechanisms^51^.

However, light quality from the total solar eclipse differs from that observed during dusk, with variable transmissivity of blue and red spectrums^52^. Spectral variations for inducing growth or long distance signaling is mitigated through protein and transcriptome interactions stimulated under dynamic light conditions^53,54^. Here, rapid changes in light conditions induced by the solar eclipse infers subsequent changes in photoreception gene regulations (e.g., early light-inducible proteins (ELIPs)), similarly to those observed in light stress conditions^53,55,56^. It is recognized that leaves in the chamber did not directly experience the solar eclipse because the artificial light source only emulates ambient PPFD inside the chamber. Reproducing red and blue transmissivity rates while measuring fluorescence dark-acclimation are not currently possible. Additionally, temperature and humidity conditions can produce substantial errors of gas exchange rates altering stomatal responses and VPD at the leaf-level^44,57^. Repeated saturating pulses under dynamic actinic light on the same leaf was considered to have negligible effects on fluorescence measurements as duration between pulses was longer (i.e., every 30 seconds) than rapid light fluorescence curves measurements (e.g., 0.8–10 seconds)^58,59^. Nevertheless, Ψ_*L*_ and time point fluorescence measurements were taken under direct influence of the spectral variations of the eclipse and were not significantly different from chamber observations (Fig. 3b). Leaf-hydraulic and photochemical parameters observed during the eclipse differed from dusk conditions, indicating that the physiological responses observed were a consequence of the solar eclipse. The eclipse prompted similar dark-acclimation rates of both hydraulic and photochemical traits, while Ψ_*L*_ lagged *F*_*v*_*’*/*F*_*m*_*’* recovery at dusk the prior night (Fig. S2). Recovery during the eclipse was likely stimulated by simultaneous reductions in radiation and VPD.

The duration of only two minutes for totality was not sufficient to restore dark-acclimation state aligning with abundant evidence that longer periods (up to 15 minutes) are required to fully open reaction centers and NPQ to relax^60,61^. NPQ is a powerful photo-protective mechanism that turns on rapidly at high light intensity but turns off more slowly upon a return to limit irradiance, especially in natural settings, with consequent reduction in quantum yield of photosynthesis and slow recovery to dark acclimated state^43,62^. After totality, *F*_*v*_*’*/*F*_*m*_’ deviated from the linear recovery to dark-acclimation response. Time series measurements of fluorescence parameters during the eclipse provided insight on photoperiod stress that were validated with the time point measurements (Fig. 3a).

Rapid fluctuations of radiation intensity and availability are common in all ecosystems through clouds diffusing light and sunflecks. However, shrub communities rarely experience self-induced shading as canopy architecture typically promotes high leaf exposure, maximizing radiation throughout the canopy^28,63^. Under consistent light and environmental conditions, carbon assimilation and stomatal conductance are independently regulated via endogenous clocks^16,64^. Additionally, photosynthetic pigments and chlorophyll fluorescence oscillate corresponding to cellular/tissue specific clocks reducing photochemical damage^16,31,65^. The short-term stress response here observed likely stems from, albeit not directly tested, disruptions of circadian clock (Fig. 3)^66^. Our research further elucidates gaps that need to be filled in the importance of coupling physiological mechanisms and endogenous clock interactions when conducting field-based observations^16^.

The unexpected drop in radiation and evaporative demand observed during the eclipse prompted biophysical and physiological regulation of stomata, minimizing the risk of cavitation under suboptimal conditions for carbon assimilation. Light limitations and reduced stomatal conductance consequently constrained carbon assimilation. Desert shrubs are typically not light limited but rather limited by leaf-water supply^30^. Throughout the day of the eclipse, maximum assimilation rates (e.g., 1.9 *μmol* CO_2_ *m*^−2^*s*^−1^) of sagebrush were less than half of early spring rates from the great basin under full light (4.3 *μmol* CO_2_ *m*^−2^*s*^−1^) suggesting substantial soil-water limitations^67^. Low stomatal conductance (i.e., 0.012 *mol* H_2_O *m*^−2^*s*^−1^) prior to the eclipse and the strong correlation between VPD and stomatal closure further supports co-limitations by soil and atmospheric drought in big sagebrush^13^.

During the eclipse, guard cell closure reduced hydraulic strain on leaf-xylem conduits and extra-xylery pathways within the leaf in response to low light conditions coupled with high VPD. This is a characteristic response of drought to minimize water loss^13,68,69^ and stomatal closure may be fast in zero light under atmospheric drought^70^ or soil drought^71^. Soil-water limitations observed with predawn leaf water potentials (i.e., −2.0 MPa) for both days of measurement are consistent with low gas exchange rates (Table 1, Fig. S1)^72,73^. Leaf-water potential and hydraulic response at dusk and directly following totality were significantly different (i.e., Ψ_*L*_) with dusk being 16.0% lower than following totality even though net radiation was approximately equal for each corresponding period (Fig. S2, Table 1). This lends support to the idea that hydraulic tension is not driven solely by stomatal conductance and light availability, but rather consequent of evaporative demand^74–77^.

Diminishing radiation caused declines in transpiration and recovery of leaf water potentials, which allowed for inference of whole-plant conductivity without measuring predawn hydraulic status. Quantifying *K*_*L*_ with the first derivative of temporal variations in transpiration rates and hydraulic strain within xylem and extra-xylery pathways provides a more robust hydraulic estimate^78^. This is especially true when assumptions of Darcy’s law approximations for *K*_*L*_ are not met, such as no nighttime transpiration, negligible stem capacitance, and homogeneous leaf water potentials throughout the canopy^79^. During the course of measurements, evaporative demand was lower at predawn though VPD remained high (i.e., greater than 0.5 kPa), violating the assumption of no night transpiration, thus requiring us to exclude predawn Ψ_*L*_ when estimating *K*_*L*_ (Fig. 2). Finally, stem capacitance was assumed to be negligible due to measurements being taken directly on leaves and the small amount of conducting xylem in big sagebrush^80^.

**Estimating regional reductions of carbon assimilation** during the eclipse resulted in a 14% decrease when compared to modeled assimilation across the identical leaf area under no eclipse conditions (Fig. 4). Modeling carbon assimilation throughout the entire range of sagebrush subspecies within the western United States under the *umbra* was done with consideration of late-season gas exchange rates having less variation under drought conditions^73,81^. Sagebrush communities, like other desert shrubs, allow for relatively simple estimation of the eclipse effects because most populations rarely experience light limitations during the day. Additionally, big sagebrush canopy traits are known to maximize water transport per unit leaf area under drought conditions through high whole-plant conductivity, increased specific leaf area, and deep rooting^13,29,30,82^.

Estimating carbon uptake across large ecosystem areas requires many assumptions and thus a high degree of uncertainty. Linear big-leaf scaling approaches assume a linear response in assimilation rates within the canopy and spatially across the landscape^83–85^. The modeled assimilation rates of big sagebrush for this study were assumed to have minimal variation as shrub canopy structure limits self-shading and maximizes radiation inputs throughout the entire canopy^28,30^. Unfortunately, this is likely not true due to cloud cover, variations of air quality influencing diffuse light (e.g., wildfire smoke and air pollution), and because of temporal variations in radiation at time of the solar eclipse. Furthermore, our estimate of daily carbon assimilation assumes that the eclipse passed all sagebrush communities at the same time and under same environmental conditions. Despite these limitations, our estimated assimilation reduction is comparable to the observed 25% decrease in sapflow of beech trees during the 1999 solar eclipse^19^.

Overall, the estimate of reduced carbon assimilation is conservative as effects of the partial eclipse were not included due to uncertainty of light availability within the penumbra based on proximity to totality and topography. Further, other shrubs and vegetation within the path of totality were specifically excluded in the calculations for the reductions of assimilation. The 5.5 × 10^−6^ Pg reduction of carbon fixation from big sagebrush is substantial, but is a negligible reduction in comparison to global terrestrial carbon assimilation rates of 146.1 ± 21.3 Pg of carbon per year^86^. Nevertheless, scaling leaf-level gas exchange serves as a proxy for the magnitude of carbon dynamics on regional scales and big sagebrush dominated communities are least likely to violate stated assumptions in ways that fundamentally change the calculated reductions in carbon assimilation.

The uniqueness of a solar eclipse has allowed us to test whether a short-term near darkness causes similar physiological responses to dawn and dusk as found in animals. We observed stress response to photochemical pathways disrupted by diminishing and recovery of midday incoming radiation on photosynthesis that cannot be predicted from changes in PPFD alone. Incorporating metrics of photochemistry and plant-water processes provide a more holistic understanding on the physiological responses to light stress. Additionally, validating mechanisms of plant response to *in situ* changes in radiation and VPD are similar to response curve approaches to those from equivalent environmental drivers.

## Methods

### Site description

The site for field measurements was located within the path of totality near the line of maxima (43 °31′53.71 N, 109 °28′20.21 W, 2073 m.a.s.l.) in northwestern Wyoming approximately 80 km southeast of Yellowstone National Park. Local vegetation is dominated by mountain big sagebrush (*Artermisia tridentata* ssp. *vaseyana*) with sporadic presence of xeric shrubs, grasses, and annual forbs. Mean annual precipitation of the area is 550 mm. The experimental site experienced 2 minutes and 18 seconds of totality, with a total duration of the partial and total solar eclipse of 2 hours 45 minutes and 36 seconds.

### Field Data Collection

A 2 m tall micrometeorological tower was deployed one-day prior to the solar eclipse, recording net radiation using a four-channel net radiometer (CNR-4, Kipp and Zonen, Delft, Netherlands), air temperature and humidity (HC2A, Rotronic, Hauppauge, NY), barometric pressure (PtB110, Vaisala, Helsinki, Finland), and wind speed and direction (05103, Young, Traverse City, MI). Data were sampled in 5-second intervals and 60-second averages were recorded on a CR3000 datalogger (Campbell Scientific, Logan, UT).

Individual time point measurements of leaf-water potentials and chlorophyll *a* fluorescence were made on 6 replicates using the pressure chamber method (PMS Instrument Company, Albany, Oregon) and a hand-held fluorometer (PSI Drasov, Czech Republic), respectively. Leaf water potential and fluorescence measurements were taken the day prior to the eclipse (August 20, 2017) to quantify diel patterns (05:00, 10:00, 15:15, 19:25 MST). On the day of the eclipse (August 21, 2017), Ψ *L* measurements were taken at predawn (05:00 MST), 30 minutes prior to totality (10:00 MST), at the end of totality (10:40 MST), 30 minutes post totality (11:25 MST), and approximately 30 minutes post partial eclipse (12:30 MST). Hand-held fluorometer measurements occurred simultaneously to Ψ_*L*_ measurements, except during totality (10:40 MDT) due to time constraints resulting from the short duration of totality and harvesting time. Actinic light tracked ambient light conditions with saturation pulses higher than 2,000 *μmol* photons m^−2^*s*^−1^ for calculating fluorescence parameters. Non-photochemical quenching (NPQ) was calculated using dark-acclimated maximal chlorophyll fluorescence (*F*_*m*_) measured with the same hand-held fluorometer 30 minutes prior to sunrise the day of the eclipse.

Leaf-level gas exchange was measured on a big sagebrush individual using an infrared gas analyzer (IRGA) LI-6400XT portable photosynthesis system (LiCor Biosciences, Lincoln, NE). Light response curves were taken on the morning of the eclipse. Photosynthesis (*A*_*L*_), transpiration (*E*_*L*_), stomatal conductance ((*g*_*s*_)), and chlorophyll *a* fluorescence (*F*_*v*_*’*/*F*_*m*_’) were collected every 30 seconds from 8:18 am to 13:19 MST. During the eclipse, measuring light tracked ambient light conditions with saturation pulses greater than 2,000 *μmol* photons m^−2^*s*^−1^. Leaf chamber settings were maintained at a flow rate of 300 *μmol s*^−1^, 400 ppm CO_2_, relative humidity was maintained at 50.9 ± 10.4%, and leaf temperature maintained at 21.4 ± 1.7 °C. A photosynthetic photon flux density (PPFD) sensor attached to the analyzer head tracked incoming radiation for the entire duration measurements and an integrated leaf chamber fluorometer (model 6400-40, LiCor Biosciences, Lincoln, NE) was used to reproduce ambient light conditions inside the chamber, through the duration of the eclipse.

### Data analysis

Whole-plant conductivity (*K*_*L*_) was estimated from Ψ_*L*_ and *E*_*L*_ rates according to Darcy Law approximations^76,78^. Darcy Law approximations assume predawn Ψ_*L*_ are in equilibrium with soil water potential, representing a minimal pressure prior to transpiration^78,87^. Before sunrise, VPD remained elevated (>0.5 kPa) creating uncertainty on equilibration of predawn Ψ_*L*_; thus, linear regression methods of diel relationships of transpiration and Ψ_*L*_ were considered to be more robust than the change between predawn and midday pressures^88^. Photosystem II efficiency (PSII) was partitioned into PPFD intervals of 0, 400, 800, and 1200 *μmol m*^−2^*s*^−1^ with a ±25 *μmol* m^−2^*s*^−1^ window before and after totality. To account for autocorrelation, random samples (*n* = 4) were selected for each interval and statistically assessed using t-tests between interval-pairs from pre- and post-totality windows of the eclipse.

Data from leaf-level photosynthesis measurements were multiplied by the allometric leaf area using mapped distributions of big sagebrush and the temporal position of the umbra at one-minute intervals across totality through Oregon, Idaho, and Wyoming. We acknowledge there is inevitable uncertainty of gas exchange rates when up scaling vast areas of the continent. One assumption of the estimated species distribution is that all three subspecies (A. tridentata ssp. *vaseyana*, spp. *tridentata*, spp. *wyomingensis*) of big sagebrush use the same allometric relationships. Moreover, we also assumed that the late time period in the growing season caused all of the different subspecies to show similar soil water limitations and thus similar transpiration rates and hydraulic properties^73^. Specifically, Kolb and Sperry (1999) observed no significant difference in gas exchange rates between sagebrush subspecies as soil water potential decrease (i.e., late growing season) imposed by hydraulic limitations within the xylem. Gas exchange values observed during the eclipse were comparable to other late growing season aproximately 0.4 to 1.03 *μmol* CO_2_ *m*^−2^*s*^−1^ ^89^. An additional assumption is that our leaf gas exchange data was representative of the average leaf within a sagebrush canopy, which is strongly supported by literature synthesis^30^. Nebraska was excluded from the estimated big sagebrush species distribution due to insufficient spatial resolution. Spatial products included 90 m state-level big sagebrush data products from 1996–2001^90^ and 30 m shrub distribution derived in 2011 from NLCD community data^91^.

Distribution maps for sagebrush and shrub densities were masked to the umbra position in 1-minute intervals. Big sagebrush and shrub areas were calculated from the masked umbra and a linear regression approach between the coarse-scale big sagebrush distribution raster (i.e., 90 m). A fine-scale shrub distribution raster (i.e., 30 m) was used to differentiate shrubs from sagebrush on the finer resolution map. The resulting raster map contained big sagebrush as a binary coverage of 50% or greater. The uncertainty of absolute distribution in each pixel was inferred to be 50% to 100% coverage, as sagebrush densities less than 50% of pixel area are not registered. Big sagebrush stand densities were calculated from allometric relationships of 20- and 45- years old stands, with 24% and 54% ground coverage respectively^92^. We note that those sagebrush allometric relationships were not different from global shrub relationships and that hydraulic relationships of shrub allocation to leaves and stems are different from other woody plants^30^ giving confidence that the scaling approach works with other shrubs. Leaf area index (LAI) parameters for scaling leaf-level gas exchange measurements to big sagebrush distributions within the region were assumed to be 0.51 to 1.2 *m*^2^*m*^−2^ based on literature values from 20 to 39 years old stands^93^. Light response was estimated using Marshall and Biscoe^94^ by means of changes in ambient radiation during the eclipse. The reduction of carbon assimilation was then calculated by differencing the predicted and observed photosynthetic rates throughout the duration of the eclipse. This scaling approach does not account for individual nor regional variability of leaf-level water potentials or photochemcial efficiencies. Statistical tests, data modeling and scaling were conducted using R (Version 3.31). Package source file and script are available on GitHub (https://github.com/dbeverly/GreatAmericanEclipse). Data is accessible through University of Wyoming Data Corral (https://datacorral.uwyo.edu/).

## Supplementary information

Hydraulic and photosynthetic responses of big sagebrush to the 2017 total solar eclipse: Supplementary information

## References

[1] Miller, J. D. Americans and the 2017 Eclipse: An initial report on public viewing of the August total solar eclipse. *Tech. Rep*., Ann Arbor: University of Michigan (2017).

[2] Fred Espenak. Total Solar Eclipse of 2017 Aug 21 (2018).

[3] Brown T, Brown K (2017). In the shadow of the Moon, what type of solar eclipse will we see?. Sci. Activities: Classr. Proj. Curriculum Ideas.

[4] Elgar S (2017). Solar energy: Switch it off on eclipse day. Nature.

[5] Uetz GW (1994). Behavior of Colonial Orb-weaving Spiders during a Solar Eclipse. Ethology.

[6] Tramer EJ (2000). Bird Behavior During a Total Solar Eclipse. The Wilson Ornithol. Soc..

[7] Galen, C. *et al*. Pollination on the Dark Side: Acoustic Monitoring Reveals Impacts of a Total Solar Eclipse on Flight Behavior and Activity Schedule of Foraging Bees. *Annals Entomol. Soc. Am*., 10.1093/aesa/say035 (2018).

[8] Branch JE, Gust DA (1986). Effect of solar eclipse on the behavior of a captive group of chimpanzees (Pan troglodytes). American Journal of Primatology.

[9] Rutter S, Tainton V, Champion R, Le Grice P (2002). The effect of a total solar eclipse on the grazing behaviour of dairy cattle. Applied Animal Behaviour Science.

[10] Farquhar GD, Sharkey TD (1982). Stomatal Conductance and Photosynthesis. Annual Review of Plant Physiology.

[11] Krause GH, Weis E (1991). Chlorophyll Fluorescence and Photosynthesis: The Basics. Annual Review of Plant Physiology and Plant Molecular Biology.

[12] Baker NR (2008). Chlorophyll Fluorescence: A Probe of Photosynthesis *In Vivo*. Annual Review of Plant Biology.

[13] Naithani KJ, Ewers BE, Pendall E (2012). Sap flux-scaled transpiration and stomatal conductance response to soil and atmospheric drought in a semi-arid sagebrush ecosystem. Journal of Hydrology.

[14] Guanter L (2014). Global and time-resolved monitoring of crop photosynthesis with chlorophyll fluorescence. Proceedings of the National Academy of Sciences of the United States of America.

[15] Martin-StPaul N, Delzon S, Cochard H (2017). Plant resistance to drought depends on timely stomatal closure. Ecology Letters.

[16] Resco de Dios V, Gessler A (2017). Circadian regulation of photosynthesis and transpiration from genes to ecosystems. Environmental and Experimental Botany.

[17] Yarkhunova Y (2018). Circadian rhythms are associated with variation in photosystem II function and photoprotective mechanisms. Plant, Cell & Environment.

[18] Häberle K-HH, Reiter I, Patzner K, Heyne C, Matyssek R (2001). Switching the light off: A break in photosynthesis and sap flow of forest trees under total solar eclipse. Meteorologische Zeitschrift.

[19] Steppe K, Lemeur R, Samson R (2002). Sap flow dynamics of a beech tree during the solar eclipse of 11 August 1999. Agricultural and Forest Meteorology.

[20] Economou G (2008). Eclipse effects on field crops and marine zooplankton: The 29 March 2006 total solar eclipse. Atmospheric Chemistry and Physics.

[21] Tominaga J (2010). Eclipse Effects on CO 2 Profile within and above Sorghum Canopy. Plant Production Science.

[22] Knick ST (2003). Teetering on the edge or too late? Conservation and research issues for avifauna of sagebrush habitats. The Condor.

[23] Bradley BA (2009). Regional analysis of the impacts of climate change on cheatgrass invasion shows potential risk and opportunity. Global Change Biology.

[24] Rottler CM, Burke IC, Palmquist KA, Bradford JB, Lauenroth WK (2018). Current reclamation practices after oil and gas development do not speed up succession or plant community recovery in big sagebrush ecosystems in Wyoming. Restoration Ecology.

[25] McArthur, E. D. & Ott, J. E. Potential natural vegetation in the 17 conterminous western United States. *UNITED STATES DEPARTMENT OF AGRICULTURE FOREST SERVICE GENERAL TECHNICAL REPORT INT* 16–28 (1996).

[26] Lorenz K, Lal R (2005). The Depth Distribution of Soil Organic Carbon in Relation to Land Use and Management and the Potential of Carbon Sequestration in Subsoil Horizons. Advances in Agronomy.

[27] Knapp AK (2008). Shrub encroachment in North American grasslands: shifts in growth form dominance rapidly alters control of ecosystem carbon inputs. Global Change Biology.

[28] Neufeld HS (1988). Conopy architecture of Larrea tridentata (DC.) Cov., a desert shrub: foliage orientation and direct beam radiation interception. Oecologia.

[29] Caldwell MM, Richards JH (1989). Hydraulic lift: water efflux from upper roots improves effectiveness of water uptake by deep roots. Oecologia.

[30] Mencuccini M (2003). The ecological significance of long-distance water transport: Short-term regulation, long-term acclimation and the hydraulic costs of stature across plant life forms. Plant, Cell and Environment.

[31] García-Plazaola JI (2017). Endogenous circadian rhythms in pigment composition induce changes in photochemical efficiency in plant canopies. Plant, Cell & Environment.

[32] Dodd IC (2005). Root-To-Shoot Signalling: Assessing The Roles of â€˜Up’ In the Up and Down World of Long-Distance Signalling In Planta. Plant and Soil.

[33] Hotta CT (2007). Modulation of environmental responses of plants by circadian clocks. Plant, Cell & Environment.

[34] Dakhiya Y, Hussien D, Fridman E, Kiflawi M, Green R (2017). Correlations between Circadian Rhythms and Growth in Challenging Environments. Plant physiology.

[35] Shalit-Kaneh A, Kumimoto RW, Filkov V, Harmer SL (2018). Multiple feedback loops of the Arabidopsis circadian clock provide rhythmic robustness across environmental conditions. Proc. Natl. Acad. Sci. United States Am..

[36] Price J, Laxmi A, Martin SKS, Jang J-C (2004). Global Transcription Profiling Reveals Multiple Sugar Signal Transduction Mechanisms in Arabidopsis. The Plant Cell.

[37] Sami F, Yusuf M, Faizan M, Faraz A, Hayat S (2016). Role of sugars under abiotic stress. Plant Physiology and Biochemistry.

[38] Zhu J-K (2016). Abiotic Stress Signaling and Responses in Plants. Cell.

[39] Dow GJ, Berry JA, Bergmann DC (2017). Disruption of stomatal lineage signaling or transcriptional regulators has differential effects on mesophyll development, but maintains coordination of gas exchange. New Phytologist.

[40] Osmond, B. C. What is photoinhibition? Some insights from comparisons of shade and sun plants. *Photoinhibition Photosynth. : from Mol. Mech. to Field* 1–24 (1994).

[41] Horton P, Ruban AV, Walters RG (1996). Regulation of Light Harvesting in Green Plants. Annual Review of Plant Physiology and Plant Molecular Biology.

[42] Demmig-Adams B, Adams WW (2006). Photoprotection in an ecological context: the remarkable complexity of thermal energy dissipation. New Phytologist.

[43] Ruban AV (2016). Nonphotochemical Chlorophyll Fluorescence Quenching: Mechanism and Effectiveness in Protecting Plants from Photodamage. Plant Physiology.

[44] Long SP, Bernacchi CJ (2003). Gas exchange measurements, what can they tell us about the underlying limitations to photosynthesis? Procedures and sources of error. Journal of Experimental Botany.

[45] Demmig-Adams B, Adams WW (1996). Xanthophyll cycle and light stress in nature: uniform response to excess direct sunlight among higher plant species. Planta.

[46] Chaves MM, Maroco JP, Pereira JS (2003). Understanding plant responses to drought - From genes to the whole plant. Functional Plant Biology.

[47] Bailleul B (2010). An atypical member of the light-harvesting complex stress-related protein family modulates diatom responses to light. Proc. Natl. Acad. Sci. United States Am..

[48] Schumann T, Paul S, Melzer M, Dörmann P, Jahns P (2017). Plant Growth under Natural Light Conditions Provides Highly Flexible Short-Term Acclimation Properties toward High Light Stress. Front. Plant Sci..

[49] Liepert BG (2002). Observed reductions of surface solar radiation at sites in the United States and worldwide from 1961 to 1990. Geophysical Research Letters.

[50] Xu P, Tian L, Kloz M, Croce R (2015). Molecular insights into Zeaxanthin-dependent quenching in higher plants. Scientific Reports.

[51] Chen J-W (2016). Photosynthesis, light energy partitioning, and photoprotection in the shade-demanding species Panax notoginseng under high and low level of growth irradiance. Functional Plant Biology.

[52] Psiloglou BE, Kambezidis HD (2007). Performance of the meteorological radiation model during the solar eclipse of 29 March 2006. Atmospheric Chemistry and Physics Discussions.

[53] Adamska I, Ohad I, Kloppstech K (1992). Synthesis of the early light-inducible protein is controlled by blue light and related to light stress. Proc. Natl. Acad. Sci. United States Am..

[54] Galvão VC, Fankhauser C (2015). Sensing the light environment in plants: photoreceptors and early signaling steps. Current Opinion in Neurobiology.

[55] Heddad M, Adamska I (2000). Light stress-regulated two-helix proteins in Arabidopsis thaliana related to the chlorophyll a/b-binding gene family. Proc. Natl. Acad. Sci..

[56] Guan Z (2016). Identification and expression analysis of four light harvesting-like (Lhc) genes associated with light and desiccation stress in Ulva linza. J. Exp. Mar. Biol. Ecol..

[57] Slatyer RO (1971). Effect of errors in measuring leaf temperature and ambient gas concentration on calculated resistance to CO|2 and water vapor exchanges in plant leaves. Plant Physiology.

[58] de Sousa CAF (2017). A procedure for maize genotypes discrimination to drought by chlorophyll fluorescence imaging rapid light curves. Plant Methods.

[59] White AJ, Critchley C (1999). Rapid light curves: A new fluorescence method to assess the state of the photosynthetic apparatus. Photosynthesis Research.

[60] Schansker G, Tóth SZ, Strasser RJ (2006). Dark recovery of the Chl a fluorescence transient (OJIP) after light adaptation: The qT-component of non-photochemical quenching is related to an activated photosystem I acceptor side. Biochimica et Biophysica Acta (BBA) - Bioenergetics.

[61] Kalaji HM (2014). Frequently asked questions about *in vivo* chlorophyll fluorescence: Practical issues. Photosynthesis Research.

[62] Rohacek K (2014). Relaxation of the non-photochemical chlorophyll fluorescence quenching in diatoms: kinetics, components and mechanisms. Philos. Transactions Royal Soc. B: Biol. Sci..

[63] Brantley ST, Young DRL-A (2007). Index and Light Attenuation in Rapidly Expanding Shrub Thickets. Notes Ecology.

[64] Resco de Dios V (2017). Circadian Regulation and Diurnal Variation in Gas Exchange. Plant physiology.

[65] Gent, M. Dynamic carbohydrate supply and demand model of vegetative growth: response to temperature, light, carbon dioxide, and day length. *Agronomy* **8**, 10.3390/agronomy8020021 (2018).

[66] Guadagno CR, Ewers BE, Weinig C (2018). Circadian Rhythms and Redox State in Plants: Till Stress Do Us Part. Frontiers in Plant Science.

[67] Prater MR, Obrist D, Arnone JA, DeLucia EH (2006). Net carbon exchange and evapotranspiration in postfire and intact sagebrush communities in the Great Basin. Oecologia.

[68] Schulze E-D (1986). Carbon dioxide and water vapor exchange in response to drought in the atmosphere and in the soil. Ann. Rev. Plant Physiol.

[69] Scoffoni C (2017). Outside-xylem vulnerability, not xylem embolism, controls leaf hydraulic decline during dehydration. Plant physiology.

[70] Ogle K (2012). Differential daytime and night-time stomatal behavior in plants from North American deserts. New Phytologist.

[71] Greenham K (2017). Temporal network analysis identifies early physiological and transcriptomic indicators of mild drought in brassica rapa. eLife.

[72] DeLucia EH, Schlesinger WH (1991). Resource-Use Efficiency and Drought Tolerance In Adjacent Great Basin and Sierran Plants. Ecology.

[73] Kolb KJ, Sperry JS (1999). Differences in drought adaptation between subspecies of sagebrush (Artemisia tridentata). Ecology.

[74] Elfving DC, Kaufmann MR, Hall AE (1972). Interpreting Leaf Water Potential Measurements with a Model of the Soil-Plant-Atmosphere Continuum. Physiol. Plantarum.

[75] Mott KA, Parkhurst DF (1991). Stomatal responses to humidity in air and helox. Plant, Cell and Environment.

[76] Sperry JS, Hacke UG (2002). Desert shrub water relations with respect to soil characteristics and plant functional type. Funct. Ecol..

[77] Buckley, T. N. How do stomata respond to water status? *New Phytologist nph*. 15899, 10.1111/nph.15899 (2019). 10.1111/nph.15899 31069803

[78] Martnez-Vilalta J, Poyatos R, Aguade D, Retana J, Mencuccini M (2014). A new look at water transport regulation in plants. New Phytologist.

[79] Oren R (2001). Sensitivity of mean canopy stomatal conductance to vapor pressure deficit in a flooded Taxodium distichum L. forest: hydraulic and non-hydraulic effects. Oecologia.

[80] Meinzer FC, Johnson DM, Lachenbruch B, McCulloh KA, Woodruff DR (2009). Xylem hydraulic safety margins in woody plants: Coordination of stomatal control of xylem tension with hydraulic capacitance. Functional Ecology.

[81] Kwon H, Pendall E, Ewers BE, Cleary M, Naithani K (2008). Spring drought regulates summer net ecosystem CO2exchange in a sagebrush-steppe ecosystem. Agricultural and Forest Meteorology.

[82] Meinzer FC (2002). Co-ordination of vapour and liquid phase water transport properties in plants. Plant, Cell and Environment.

[83] Baldocchi DD, Harley PC (1995). Scaling carbon dioxide and water vapour exchange from leaf to canopy in a deciduous forest. II. Model testing and application. Plant, Cell and Environment.

[84] Leuning R, Kelliher FM, Pury DGG, Schulze ED (1995). Leaf nitrogen, photosynthesis, conductance and transpiration: scaling from leaves to canopies. Plant, Cell and Environment.

[85] Resco de Dios V, Loik ME, Smith R, Aspinwall MJ, Tissue DT (2016). Genetic variation in circadian regulation of nocturnal stomatal conductance enhances carbon assimilation and growth. Plant, Cell & Environment.

[86] Cheng L (2017). Recent increases in terrestrial carbon uptake at little cost to the water cycle. Nature Communications.

[87] Hochberg U, Rockwell FE, Holbrook NM, Cochard H (2018). Iso/Anisohydry: A Plant−Environment Interaction Rather Than a Simple Hydraulic Trait. Trends in Plant Science.

[88] Bucci SJ (2004). Functional convergence in hydraulic architecture and water relations of tropical savanna trees: from leaf to whole plant. Tree Physiology.

[89] Cleary MB, Naithani KJ, Ewers BE, Pendall E (2015). Upscaling CO<inf>2</inf>fluxes using leaf, soil and chamber measurements across successional growth stages in a sagebrush steppe ecosystem. Journal of Arid Environments.

[90] Comer, P., Kagan, J., Heiner, M. & Tobalske, C. Current distribution of sagebrush and associated vegetation in the western United States (excluding NM and AZ). Digital Map 1: 200,000 scale. USGS Forest and Rangeland Ecosystems Science Center, Boise, ID, and The Nature Conservancy, Boulder, CO (2002).

[91] Coulston JW (2012). Modeling percent tree canopy cover: a pilot study. Photogrammetric Engineering & Remote Sensing.

[92] Cleary MB, Pendall E, Ewers BE (2008). Testing sagebrush allometric relationships across three fire chronosequences in Wyoming, USA. Journal of Arid Environments.

[93] Cleary MB, Pendall E, Ewers BE (2010). Aboveground and belowground carbon pools after fire in mountain big sagebrush steppe. Rangeland Ecology and Management.

[94] Marshall B, Biscoe PV (1980). A model for C3 leaves describing the dependence of net photosynthesis on irradiance. Journal of Experimental Botany.

