# Supplementary material for: Hydraulic and photosynthetic responses of big sagebrush to the 2017 total solar eclipse
Source: Sci Rep. 2019 Jun 20;9:8839. doi: 10.1038/s41598-019-45400-y (PMC6586607; doi:10.1038/s41598-019-45400-y)
Supplement: Supplementary file 1 — Hydraulic and photosynthetic responses of big sagebrush to the 2017 total solar eclipse: Supplementary information [file 41598_2019_45400_MOESM1_ESM.pdf]

Hydraulic and photosynthetic responses of big sagebrush to the 2017 total solar eclipse: Supplementary information

Daniel P. Beverly[1,2,*], Carmela R. Guadagno[1], Mario Bretfeld[1], Heather N. Speckman[1,4], Shannon E. Albeke[3,4], and Brent E. Ewers[1,4]

[1]Department of Botany, University of Wyoming, Laramie, WY 82071, USA
[2]Water Resources, Environmental Science and Engineering, University of Wyoming, Laramie, WY 82071, USA
[3]Department of Geography and Wyoming Geographic Information Service Center, University of Wyoming, Laramie, WY 82071, USA
[4]Program in Ecology, University of Wyoming, Laramie, WY 82071, USA
[*]dbeverly@uwyo.edu

ABSTRACT

The total solar eclipse of August 21, 2017 created a path of totality ~ 115 km in width across the United States. While eclipse observations have shown distinct responses in animal behavior often emulating nocturnal behavior, the influence of eclipses on plant physiology are less understood. We investigated physiological perturbations due to rapid changes of sunlight and air temperature in big sagebrush (*Artemisia tridentata* ssp. *vaseyana*), a desert shrub common within the path of eclipse totality. Leaf gas exchange, water potential, and chlorophyll *a* fluorescence were monitored during the eclipse and compared to responses obtained the day before in absence of the eclipse. On the day of the eclipse, air temperature decreased by $6.4\,^{\circ}\mathrm{C}$, coupled with a 1.0 kPa drop in vapor pressure deficit having a 9-minute lag following totality. Using chlorophyll *a* fluorescence measurements, we found photosynthetic efficiency of photosystem II (F_v'/F_m') recovered to near dark acclimated state (i.e., 87%), but the short duration of darkness did not allow for complete recovery. Gas exchange data and a simple light response model were used to estimate a 14% reduction in carbon assimilation for one day over sagebrush dominated areas within the path of totality for the Western United States.

Supplementary Tables & Figures

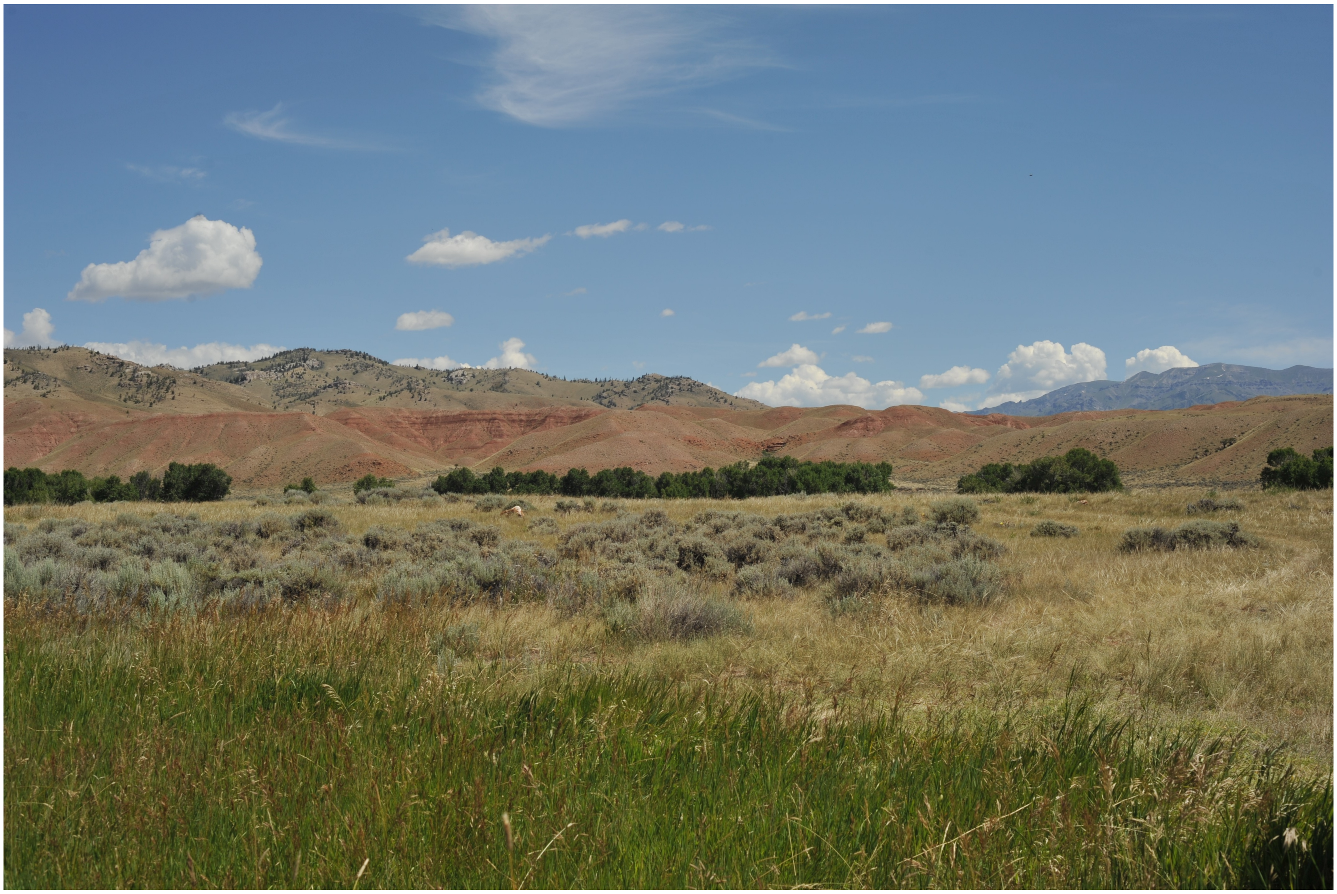

Figure 1. Picture of the sagebrush community southeast of Yellowstone National Park. Picture taken by Daniel P. Beverly.

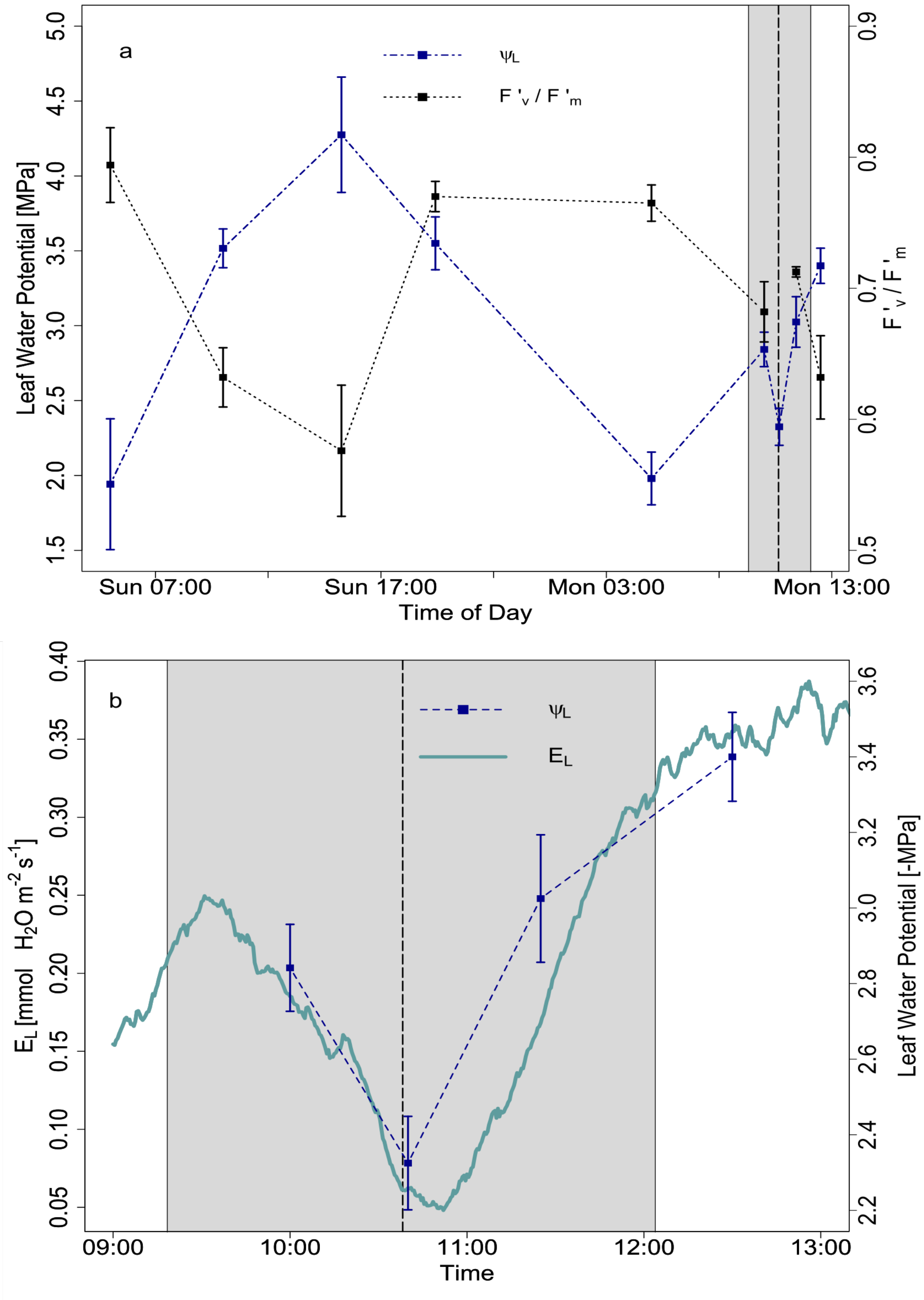

Figure 2. Two-day temporal response of mean leaf water potentials (Ψ_L) and chlorophyll *a* fluorescence (F_v' / F_m') variation represented with standard error (n = 6) (a). Transpiration (E_L) and (Ψ_L) response during the duration of the solar eclipse. Error bars are standard error (n = 6) (b). Grey shaded area representing partial solar eclipse and dotted vertical line indicates totality.

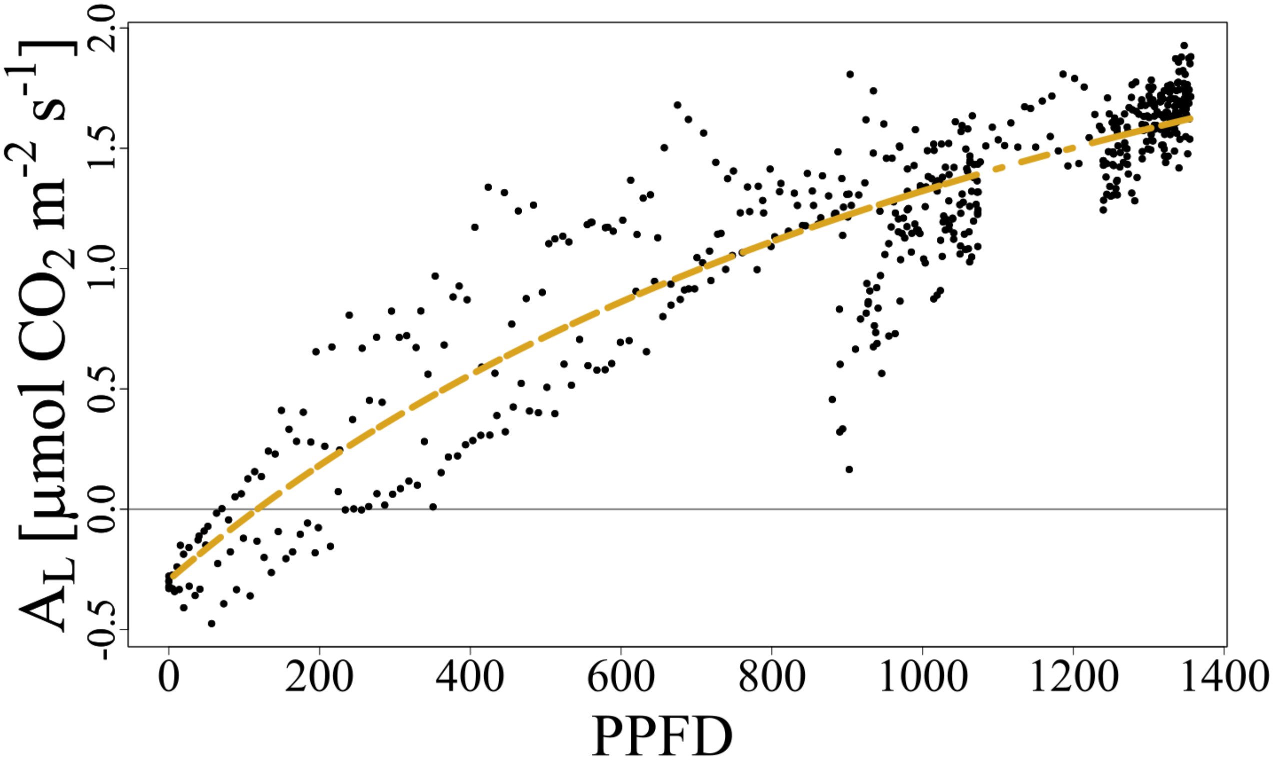

Figure 3. Response of carbon assimilation (A_L) to variable photosynthetic photon flux density (PPFD) during the solar eclipse. The points represent single measurement taken every 30 seconds while the gold line is modeled response. Reduction of A_L during diminishing light conditions responds as rapid light response curve.
